# Acetic acid stimulates G-protein-coupled receptor GPR43 and induces intracellular calcium influx in L6 myotube cells

**DOI:** 10.1371/journal.pone.0239428

**Published:** 2020-09-30

**Authors:** Hitomi Maruta, Hiromi Yamashita

**Affiliations:** 1 Department of Nutritional Science, Faculty of Health and Welfare Science, Okayama Prefectural University, Soja, Okayama, Japan; 2 Graduate School of Health and Welfare Science, Okayama Prefectural University, Soja, Okayama, Japan; University of Minnesota Medical School, UNITED STATES

## Abstract

Short chain fatty acids (SCFAs) produced endogenously in the gut by bacterial fermentation of dietary fiber have been studied as nutrients that act as signaling molecules to activate G-protein coupled receptors (GPCRs) such as GPR41 and GPR43. GPR43 functioning involves the suppression of lipid accumulation and maintaining body energy homeostasis, and is activated by acetic acid or propionic acid. Previously, we reported that the orally administered acetic acid improves lipid metabolism in liver and skeletal muscles and suppresses obesity, thus improving glucose tolerance. Acetic acid stimulates AMP-activated protein kinase (AMPK) through its metabolic pathway in skeletal muscle cells. We hypothesized that acetic acid would stimulate GPR43 in skeletal muscle cells and has function in modulating gene expression related to muscle characteristics through its signal pathway. The objective of the current study was to clarify this effect of acetic acid. The *GPR43* expression, observed in the differentiated myotube cells, was increased upon acetic acid treatment. Acetic acid induced the intracellular calcium influx in the cells and this induction was significantly inhibited by the *GPR43*-specific siRNA treatment. The calcineurin molecule is activated by calcium/calmodulin and is associated with proliferation of slow-twitch fibers. Calcineurin was activated by acetic acid treatment and inhibited by the concomitant treatment with *GPR43*-siRNA. Acetic acid induced nuclear localization of myocyte enhancer factor 2A (MEF2A), peroxisome proliferator-activated receptor γ coactivator-1α (PGC-1α), and nuclear factor of activated t cells c1 (NFATc1). However, these localizations were abolished by the treatment with *GPR43*-siRNA. It was concluded that acetic acid plays a role in the activation of GPR43 and involves the proliferation of slow-twitch fibers in L6 skeletal muscles through the calcium-signaling pathway caused by induction of intracellular calcium influx.

## Introduction

Prevalence of obesity and obesity-linked type 2 diabetes are worldwide health concerns that need urgent attention and solution. According to the International Diabetes Federation Diabetes Atlas, there were 463 million people living with diabetes globally in 2019. This number is expected to rise to 700 million by 2045 [1]. Dietary supplementation with SCFAs such as acetic acid focuses on preventing obesity and life-style related diseases [2–4]. Acetic acid significantly reduces body weight gain, suppresses liver lipogenesis and lipid accumulation in white adipose tissues, improves glucose tolerance, and protects against fatty liver [5–8].

It was demonstrated in our previous study that acetic acid stimulates the AMPK via an increase in the AMP/ATP ratio through its metabolic pathway, induces myoglobin and glucose transporter 4 (GLUT4) expressions, stimulates glucose incorporation, and suppresses lipid accumulation in the L6 skeletal muscle cells [6,9].

SCFAs administered in clinical trials show a positive effect on the ulcerative colitis Crohn’s disease, diarrhea, and obesity [10,11]. SCFAs are produced in gut by bacterial fermentation and act as signaling molecules to activate G-protein coupled receptors GPR41 and GPR43. As compared to propionic acid and butyric acid, acetic acid activates GPR43 with a higher potency than that seen in GPR41 [12,13]. GPR43 is reported to couple to Gi/o and Gq/_11_ of G proteins [12–14], which interact with several downstream molecules, and activates the pathways. It is expressed in adipose tissues, intestine, and immune cells [2,4,13–15]. GPR43 promotes the secretion of glucagon-like peptide 1 (GLP-1) in mixed colonic primary cultures and STC-1 cells [3,16]. GPR43 expressed in white adipose tissues is involved in the inhibition of fat accumulation and plays a key role in regulation of life-style related diseases [2]. However, the physiological functions of GPR43 in skeletal muscle cells have not been reported so far. We hypothesized that acetic acid would stimulate GPR43 in skeletal muscle cells and has function in modulating gene expression related to muscle characteristics through its signal pathway.

In this study, we emphasize on the function of acetic acid as an activator of GPR43 in L6 skeletal muscle cells. We reveal that acetic acid functions in the induction of calcium influx in L6 cells and increases expressions of several genes associated with slow-twitch fibers through the activation of GPR43.

## Materials and methods

### Materials

Rat L6 myoblasts (JCRB9081) were purchased from JCRB cell bank (Osaka, Japan). Dulbecco’s modified eagle medium (DMEM) was purchased from Sigma-Aldrich (MO, USA), fetal bovine serum (FBS) was purchased from Biosera (MO, USA), while the horse serum (HS) and penicillin/streptomycin were bought from Gibco (MA, USA). We procured 0.25% Trypsin-EDTA from Santa Cruz Biotechnology (TX, USA). Antibodies against AMPKα, phosphorylated AMPKα and GLUT4 were purchased from Cell Signaling (MA, USA). Antibodies against myoglobin, MEF2A, NFATc1, phosphorylated NFATc1, calcium/calmodulin-dependent protein kinase kinase β (CaMKKβ), PGC-1α, and Sp1 were obtained from Santa Cruz Biotechnology (TX, USA), while the α-tubulin antibody, calcineurin inhibitor cyclosporine A (CsA), and PLC inhibitor YM-254890 were purchased from FUJIFILM Wako Pure Chemical Corporation (Osaka, Japan). The AMPK inhibitor adenine 9-β-D-arabinofuranoside (araA), calmodulin (CaM) inhibitor N-(6-Aminohexyl)-5-chloro-1-naphthalenesulfonamide hydrochloride (W-7), *GPR43*-siRNA, potassium cyanide, succinate, 2,6-dichloroindophenol sodium salt dihydrate (DCPIP) and decylubiquinone (DUB) were purchased from Sigma-Aldrich (MO, USA). The GPR43 agonist, (S)-2-(4-chlorophenyl) -3, 3-dimethyl-N-(5-phenylthiazol-2-yl) butanamide, was purchased from Merck Millipore (DA, Germany). The phospholipase C (PLC) inhibitor, 1-[6-[[(17β)-3-Methoxyestra-1, 3, 5(10)-trien-17-yl] amino] hexyl]-1H-pyrrole-2, 5-dione (u73122), was purchased from Merck Millipore (Cambridge, UK). Lipofectamine transfection reagents, RNAiMAX was purchased from Invitrogen (MA, USA). Sepasol RNA I super G was purchased form Nacalai Tesque (Kyoto, Japan). PrimeScript RT Reagent Kit with gDNA Eraser was purchased form Takara Bio (Shiga, Japan).

### L6 cell culture and siRNA transfection

L6 cells were cultured as previously described [9]. L6 myoblasts were grown in DMEM containing 10% (v/v) FBS, 100 units/ml penicillin, and 100 μg/ml streptomycin in 5% CO_2_ at 37°C. For myotube differentiation, the medium was changed to DMEM containing 2% (v/v) horse serum when myoblasts were 80% confluent. Myotubes were harvested 8–11 days after differentiation, and experimental procedures were initiated. Differentiated myotubes were incubated with acetic acid and/or other reagents. L6 cells cultured in growth medium were transfected with 50 nM control siRNA or rat *GPR43*-siRNA (sense, 5’-CUGCUAUUGGCGCUUUGUATT-3’ and antisense, 5’-UACAAAGCGCCAAUAGCAGTT-3’) using Lipofectamine RNAiMAX for 24–48 h according to the manufacturer’s instructions. The siRNA oligonucleotides were designed to interact with *GPR43* mRNA. The down-regulation of the *GPR43* targeted siRNA was confirmed by measuring the levels of its expression using the StepOnePlus detection system (Applied Biosystems, CA, USA).

### Animal models

Eight and 20 weeks old SD rats were used for experimental procedures related to skeletal muscles. Rats were fed on a standard laboratory rodent diet (CE-2) which was purchased from Clea (Tokyo, Japan). The care and use of the animals in this study followed the guidelines of Okayama Prefectural University and the laws and notifications of the Japanese government. All animal experiments were approved by the Animal Care and Use Committee of Okayama Prefectural University (protocol number 30–4).

### Nuclear extraction and western blotting

Nuclear extraction and western blotting were performed as described previously [9]. After separation of the cytosolic and nuclear fractions, protein concentrations of each of the extracts from nuclear, cytosolic, and total fraction were determined by Bradford assay. An aliquot (15–30 μg of protein) of each extract was used for western blotting to determine the protein content of AMPKα, phosphorylated Thr-172 AMPKα, myoglobin, GLUT4, MEF2A, PGC-1α, NFATc1, phosphorylated Ser259 NFATc1, CaMKKβ, Sp1, and α-tubulin proteins. Samples were applied to 10–15% SDS-PAGE, and immuno-blotted with the corresponding antibodies. Chemiluminescent signals were visualized and quantified with ImageQuant LAS-4000 and Multi Gauge V3.2 analyzing software (Fujifilm, Tokyo, Japan).

### Quantitative RT-PCR analysis

Total RNA was isolated with Sepasol RNA I super G and genomic DNA was isolated with extraction buffer (4 M guanidine thiocinnate, 50 mM sodium citrate, 1 M Tris). The cDNA was synthesized using PrimeScript RT Reagent Kit with gDNA Eraser according to the manufacturer’s instructions. Quantitative real-time PCR analyses were performed using the StepOnePlus with KAPA SYBR^®^ FAST qPCR Kit (Kapa Biosystems, Wilmington, MA) for quantification of the specific mRNA content. Data were normalized to β-actin mRNA and expressed relative to the untreated control cells. The oligonucleotide primers sequences used in this study are listed in Table 1.

**Table 1:** List of sequences of PCR primers.

Gene	Forward	Reverse
β-actin (*actb*)	GGAGATTACTGCCCTGGCTCCTA	GACTCATCGTACTCCTGCTTGCTG
GLUT4 (*slc2a4*)	GGGCGATTTCTCCCACATAC	CTCATGGGCCTAGCCAATG
MEF2A (*mef2a*)	ATGAGAGGAACCGACAGGTG	TATCCGAGTTCGTCCTGCTT
myoglobin (*mb*)	CTAACAGCCGGCCTACACTC	CGTGCTTCTTCAGGTCCTCT
PGC-1α (*ppargc1a*)	GACCCCAGAGTCACCAAATGA	GGCCTGCAGTTCCAGAGAGT
cytochrome C (*cycs*)	AGCGGGACGTCTCCCTAAGA	CTTCCGCCCAAACAGACCA
succinate dehydrogenase (*sdha*)	TGGGGCGACTCGTGGCTTTC	CCCCGCCTGCACCTACAACC
GPR41 (*GPR41*, *ffar3*)	CCCTGGTGCTGTAGGAGCTA	CCATCACGTTGAGGGGTAGT
GPR43 (*GPR43*, *ffar2*)	CAGAGGAGAACCAGGTGGAAG	GGCAGGGACCCCAGTAAGAA
NADH dehydrogenase 1, mitochondrial (*mt-Nd1*)	CTCCCTATTCGGAGCCCTAC	ATTTGTTTCTGCTAGGGTTG

PCR-products were run on 2% agarose gel (Nippon Gene Co., Ltd., Tokyo, Japan) containing ethidium bromide (Bio-Rad, CA, USA) in TAE (40 mM Tris-Acetate (pH 7.8), 1 mM EDTA) buffer. The signal intensities were visualized and quantified with ImageQuant LAS-4000 and Multi Gauge V3.2 analyzing software (Fujifilm, Tokyo, Japan).

### Intracellular calcium measurements

The intracellular calcium concentrations were measured by detecting the fluorescence of cells treated with a calcium-sensitive indicator, Fluo-4 AM [17]. L6 cells harvested 10 days after differentiation were re-plated in the 96-well plates (Iwaki, Tokyo, Japan) at 1.5×10^4^ cells/well for 24 h. Subsequently, the Ca^2+^ levels were determined with the use of Calcium Kit II-Fluo 4 (Dojindo, Kumamoto, Japan) using Powerscan HT (BioTek, VT, US). Briefly, cells were washed twice with non-serum medium containing 2.5 mM probenecid in 24 h after re-plating. Then cells were incubated with 4 μg/ mL Fluo-4 AM and 0.025% (w/v) Pluronic F-127 for 30 min in dark at 37°C. After washings twice with non-serum medium, cells measurement was performed on a Powerscan HT instrument with an excitation band of 485/20 nm and fluorence was measured at 528/20 nm. Baseline signals (*F*_*0*_) were recorded 5 min before the addition of each stimulus. Subsequently, continuous fluorescence measurements were performed for 20 min. Results are shown as *F/F*_*0*_ ratios after background subtraction, where *F* was the fluorescence signal intensity and *F*_*0*_ was the baseline intensity, as calculated by the average of 5 frames before stimulus application [17].

### SDH activity

SDH activity was measured using spectrophotometer (U-2900, Hitachi High-Tech Corporation., Tokyo, Japan) [18]. L6 cells were detached, washed in PBS, and centrifuged at 1,000g for 5 min at 4°C. The supernatant was discarded and the cells were resuspended in PBS. After twice wash, the cell pellet was suspended in 20 mM hypotonic potassium phosphate buffer (pH 7.5). The cell lysate was freezed in liquid nitrogen and thawed at 37°C three times. The cell lysate was added to the reaction solution (25 mM potassium phosphate buffer (pH 7.5), 1 mg/ml bovine serum albumin, 300 μM potassium cyanide, 20 mM succinate and 80 μM DCPIP), the reaction mixture was incubated at 37°C for 8 min, and the baseline was measured at 600nm for 2 min. Then, the reaction was started by adding 50 μM DUB and the decrease of absorbance at 600 nm was measured for 3 min. The activity of SDH was compared by quantifying how much the added DCPIP was consumed.

### Statistical analyses

The data values have been presented as mean ± SE. The data were regarded as normally distributed and were analyzed with one-way or two-way ANOVA, followed by the Tukey-Kramer post hoc test for multiple comparisons or analyzed with unpaired Student’s t-test for direct comparison between two groups. For analysis of [Ca^2+^]_i_ measurements, peaks (maximum *F/F*_*0*_ ratio within 20 min after stimulus) were compared using one-way ANOVA, followed by the Tukey-Kramer post hoc test for multiple comparisons [17]. P values <0.05 were considered significant. All statistical analyses were performed using Statcel 4 (OMS, Tokyo, Japan).

## Results

### Expression of *GPR43* mRNA in skeletal muscles and L6 myotube cells

**Fig 1:**
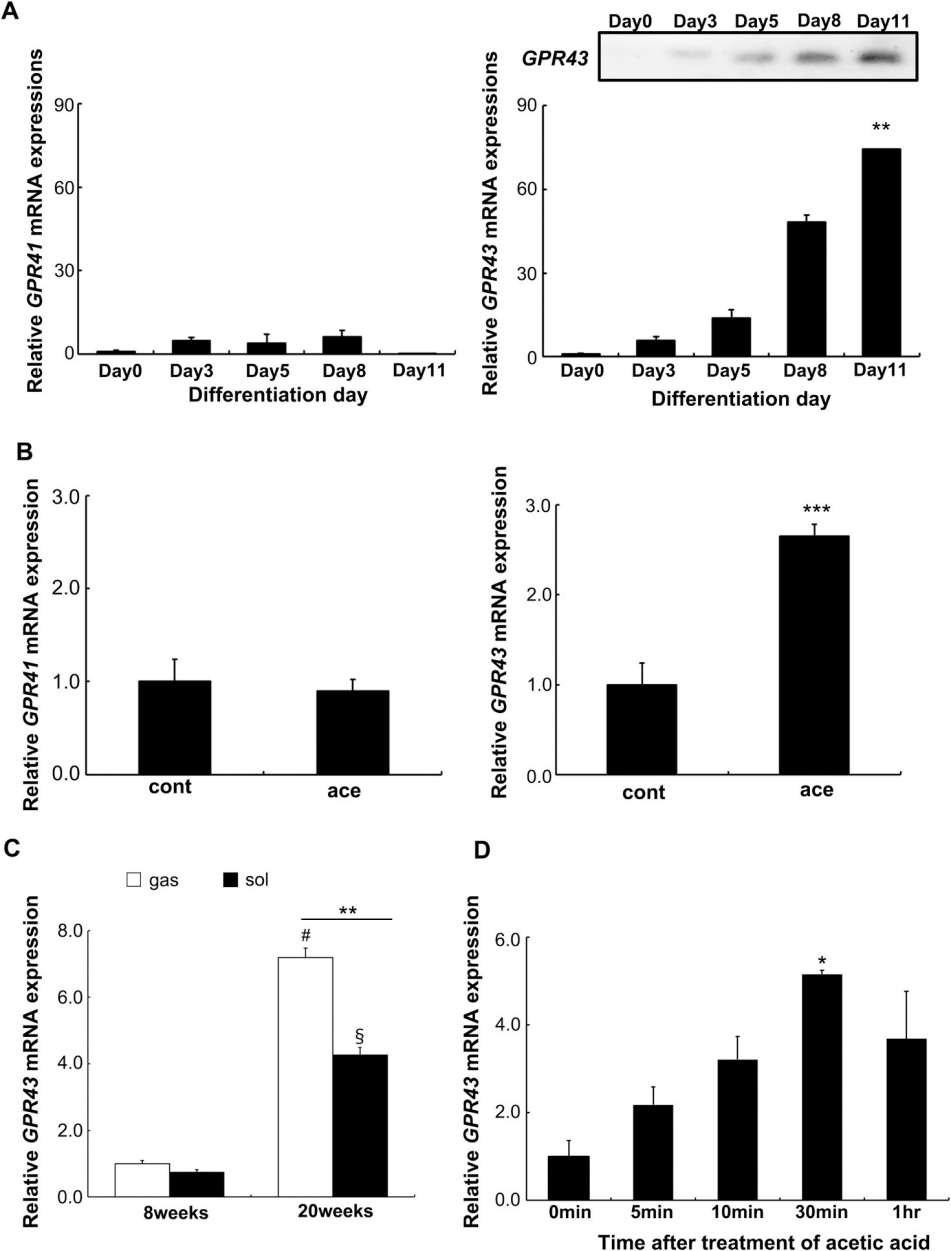
Effects of acetic acid on the expression of *GPR43*. (A) Total RNA was extracted from the differentiating L6 cells to analyze the expression of *GPR41* and *GPR43* by real-time PCR. PCR product of *GPR43* was confirmed by the electrophoresis with 2% agarose gels (insert image) Each expression level during the differentiation on each of the days was analyzed and compared among expressions of each gene. Multiple comparisons were analyzed with one-way ANOVA followed by Tukey-Kramer post hoc test. Each value is shown as the mean ± SE (n = 3–6). Statistical difference is shown as **p< 0.01, compared with day 0. (B) Expressions of *GPR41* and *GPR43* were measured in the differentiated L6 myotube cells that treated with 0.5 mM acetic acid for 30 min (ace) or untreated (cont). The statistical difference of each expression between ace and cont was analyzed using the unpaired Student’s t-test. Each value is shown as the mean ± SE (n = 3–6). Statistical difference is shown as ***p< 0.001, compared with cont. (C) Total RNA was isolated from gastrocnemius (gas) and soleus (sol) muscles of SD rats at 8 and 20 weeks of age, and the expression of *GPR43* was determined. Multiple comparisons were analyzed with two-way ANOVA followed by Tukey-Kramer post hoc test. Each value is shown as the mean ± SE (n = 3–6). Statistical differences are shown as ^#^p<0.05, compared with the expression in gas of 8 weeks; ^§^P<0.05, compared with the expression in sol of 8 weeks; **p<0.01, compared the expressions between gas and sol. (D) Differentiated L6 cells were treated with 0.5 mM acetic acid for the indicated time periods and the *GPR43* expression was analyzed. Multiple comparisons were analyzed with one-way ANOVA followed by Tukey-Kramer post hoc test. Each value is shown as the mean ± SE (n = 3–6). Statistical difference is shown as *p< 0.05, compared with 0 min.

It has been reported that *GPR41* (*ffar3*) and *GPR43* (*ffar2*) are activated by SCFAs and expressed in a large variety of tissues, including adipose tissue and gastrointestinal tract [19,20]. As shown in Fig 1, *GPR43* gene was expressed in differentiated myotube cells. The mRNA transcription was quantified in L6 myotube cells during the course of myotube differentiation until 11 days of the induction of differentiation (Fig 1A). Expression of *GPR43* was negligible in the myoblasts at day 0 though it significantly increased during differentiation (Fig 1A). On the other hand, expression of *GPR41* did not change significantly. Upon treatment with acetic acid, expression of the *GPR43* was significantly elevated whereas that of *GPR41* remained unaltered (Fig 1B). In the skeletal muscles of SD rats at 8 weeks and 20 weeks, *GPR43* was expressed in gastrocnemius and soleus muscles and the extent of expression was higher in skeletal muscles of 20 weeks old rats than that of 8 weeks old rats (Fig 1C). A time-course experiment revealed that *GPR43* was significantly increased 30 min after the treatment of acetic acid in L6 cells (Fig 1D).

### Induction of intracellular calcium influx by acetic acid in L6 cells

**Fig 2:**
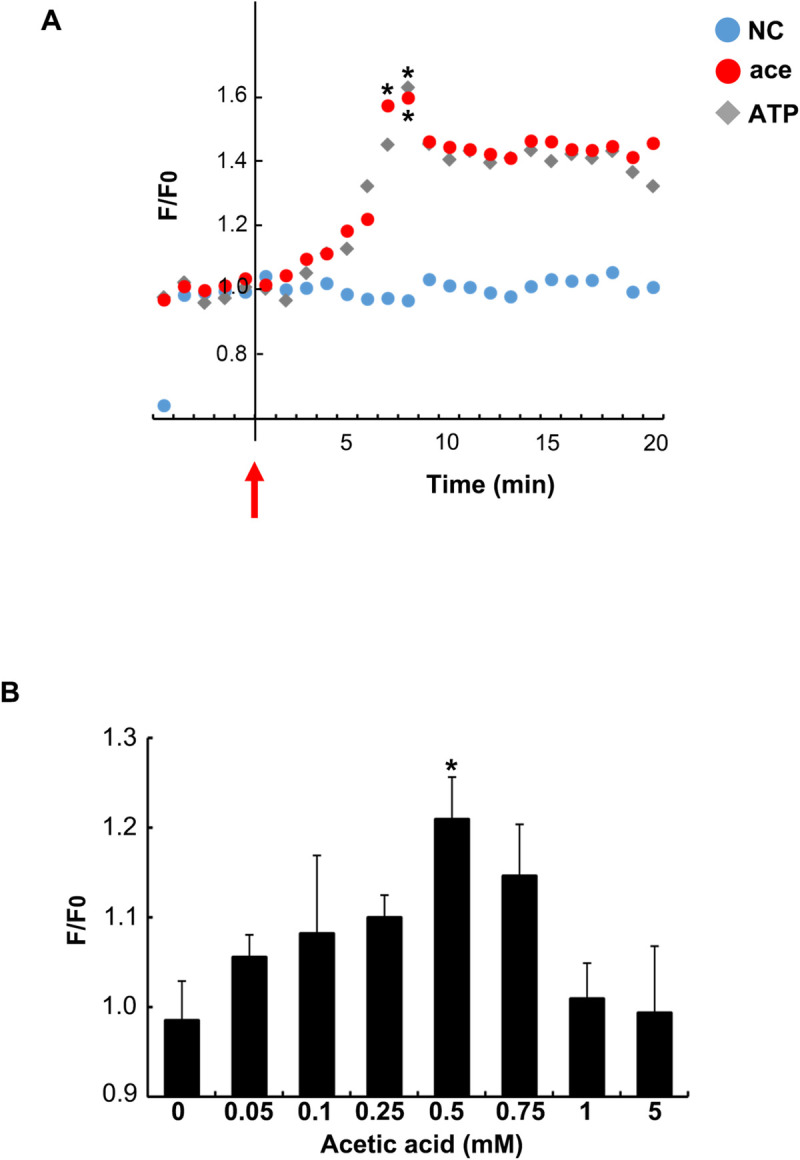
Effect of acetic acid on intracellular calcium influx. (A) Acetic acid or ATP was each added to the culture medium of differentiated L6 cells at the time points indicated with an arrow. The [Ca^2+^]i influx in L6 cells was measured after stimulation with 0.5 mM acetic acid or 25 μM ATP. Data shown are average values from three to seven independent experiments. NC: Non-stimulated condition. Peaks of average values for each treatment were analyzed with one-way ANOVA followed by Tukey-Kramer post hoc test. Statistical differences are shown as *p< 0.05 compared with NC. (B) Intracellular [Ca^2+^]i influx was measured in 8 min after the treatment of acetic acid at each of the concentrations indicated in the figure. Each bar represents the mean ± SE (n = 3–7). Multiple comparisons were analyzed with one-way ANOVA followed by the Tukey-Kramer post hoc test. Statistical differences are shown as *p< 0.05, compared with untreated (0 mM).

The intracellular calcium influx was seen in L6 cells after stimulation with acetic acid as well as 25 μM ATP. The ATP molecules induce calcium influx through the P2X receptors on the cell membrane surface [21–23]. The calcium influx was monitored for 20 min (Fig 2A). In comparison to the non-stimulated condition (NC), acetic acid and ATP stimulation significantly stimulated an increase in the intracellular calcium influx within 8 min of acetic acid treatment. Calcium influx was increased in a dose dependent manner up to the acetic acid concentration of 0.5 mM. However, addition of acetic acid in a concentration higher than 0.5 mM led to a reduction in the calcium influx. Thus, the calcium influx reached its peak at 0.5 mM acetic acid concentration (Fig 2B).

### Silencing of *GPR43* inhibits the expression of skeletal muscle related genes and proteins

**Fig 3:**
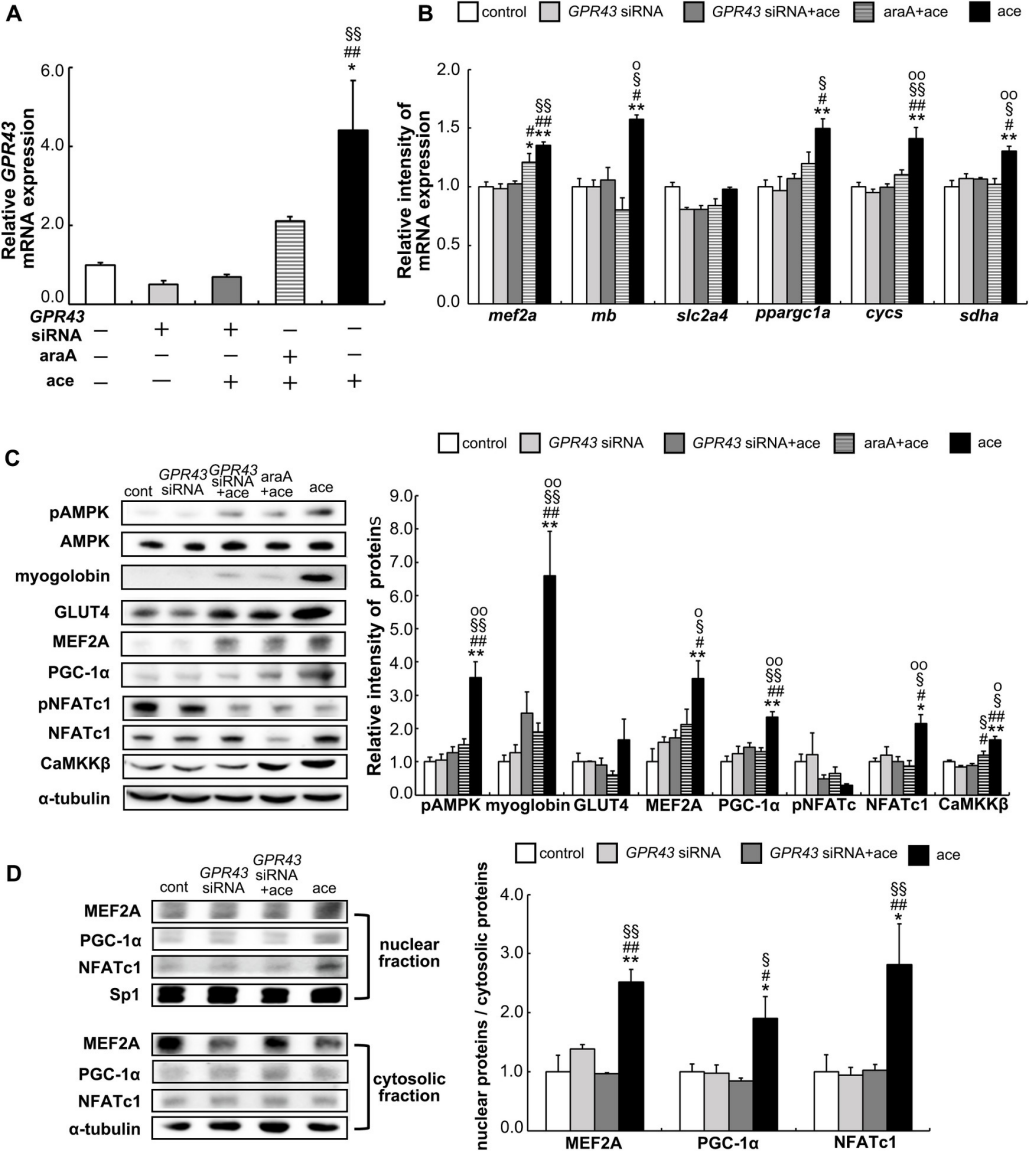
Effect of GPR43 on the expressions of myogenic genes and proteins and nuclear localization of MEF2A, PGC-1α, and NFATc1 in L6 myotube cells. L6 myotube cells were treated with 50 nM *GPR43*-specific siRNA for 24 h as described in the Materials and Methods. Total RNA was extracted from the cells after the treatment or no treatment with 0.5 mM acetic acid for 30 min and with or without 2 mM araA preincubated for 20 min. Next, quantitative real-time PCR analysis was carried out for determinations of *GPR43* gene expression (A), expressions of *mef2a*, *mb*, *slc2a4*, *ppargc1a*, *cycs*, and *sdha* genes (B). Muscle proteins, pAMPK, myoglobin, GLUT4, MEF2A, PGC-1α, pNFATc1, NFATc1, and CaMKKβ were extracted and analyzed by western blotting as described in Materials and Methods (C). Nuclear fraction and cytosolic fraction were separated from *GPR43*-specific siRNA transfected or non-transfected L6 cells after the treatment or no treatment with 0.5 mM acetic acid for 30 min. Nuclear and cytosolic proteins were examined by western blotting as described in Materials and Methods (D). Each bar represents the mean ± SE (n = 3–6). Multiple comparisons were analyzed with one-way ANOVA followed by the Tukey-Kramer post hoc test. Statistical differences are shown as*p< 0.05, **p<0.01, compared with non-treated control; ^#^p< 0.05, ^##^p<0.01, compared with *GPR43* siRNA; ^§^p< 0.05, ^§§^p<0.01, compared with *GPR43* siRNA + ace; ^o^p< 0.05, ^oo^p<0.01, compared with araA + ace.

In order to investigate the physiological role of GPR43 in L6 myoblasts, *GPR43* was knocked down by transfection of *GPR43*-specific in siRNA in the L6 cells. The inducing effect of acetic acid on the expression of *GPR43* was completely abolished by the *GPR43*-siRNA. The treatment of araA, which is an AMPK inhibitor, also showed a decline in the effect of acetic acid, but the change was not significant (Fig 3A). The expression levels of the muscle genes (*mef2a*, *mb*, *ppargc1a*, *cycs*, and *sdha*) and proteins (pAMPK, myoglobin, MEF2A, PGC-1α, NFATc1, and CaMKKβ) were elevated significantly in the acetic acid-treated cells (Fig 3B and 3C). However, there was a significant drop in the induction upon silencing of *GPR43*. On the contrary, phosphorylated NFATc1, which is inactive form, tended to decrese in the acetic acid-treated cells. In addition, araA showed a declining trend in the expressions of these genes and proteins (Fig 3B and 3C).

### GPR43 mediated nuclear localization of MEF2A, PGC-1α, and NFATc1 by acetic acid

Nuclear MEF2A, PGC-1α, and NFATc1 proteins are associated with the proliferation and generation of slow-twitch muscle fiber proteins [24–26]. We examined the impact of GPR43 on the nuclear localization of MEF2, PGC-1α, and NFATc1 upon the treatment with acetic acid. The MEF2A, PGC-1α, and NFATc1 proteins were localized in the nucleus post acetic acid treatment (Fig 3D). In contrast, their nuclear localization was drastically reduced in the cells transfected with *GPR43*–specific siRNA (Fig 3D).

### Effect of GPR43 agonist on expressions of muscle genes and proteins

**Fig 4:**
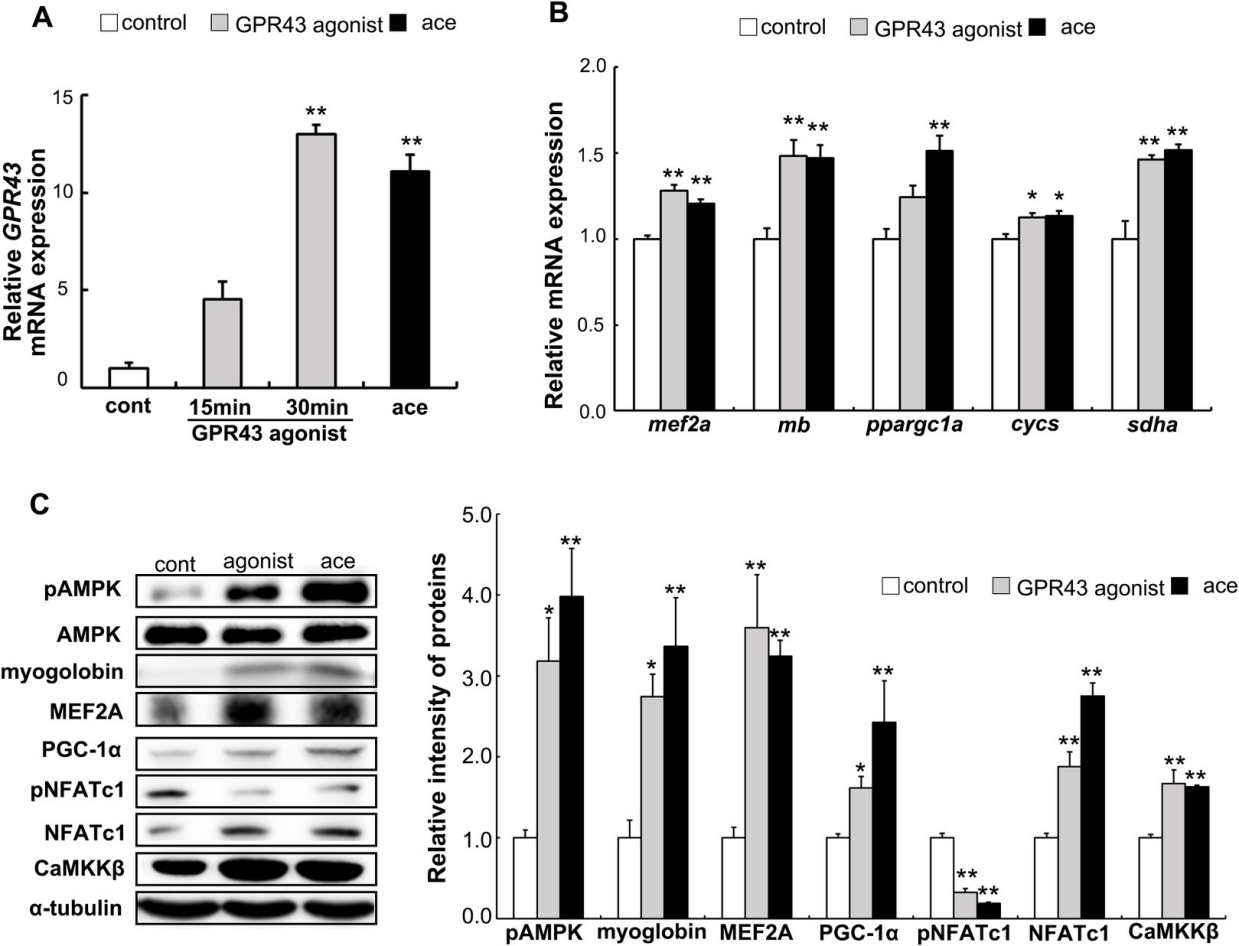
Effect of GPR43 agonist on the expressions of *GPR43*, muscle genes, and muscle proteins. Total RNA was extracted and real-time PCR analysis was carried out in order to examine the mRNA expressions of *GPR43* in the L6 myotube cells treated with 0.5 mM acetic acid for 30 min or 1.0 μM GPR43 agonist for 15 min or 30 min (A). The expressions of *mef2a*, *mb*, *ppargc1a*, *cycs*, and *sdha* genes in the L6 cells treated with 0.5 mM acetic acid or 1.0 μM GPR43 agonist for 30 min were examined by real-time PCR analysis (B). Next, muscle proteins, pAMPK, myoglobin, MEF2A, PGC-1α, pNFATc1, NFATc1, and CaMKKβ in the L6 cells treated with 0.5 mM acetic acid or 1.0 μM GPR43 agonist for 30 min were analyzed by western blotting as described in Materials and Methods (C). Each bar represents the mean ±SE (n = 3–6). Multiple comparisons were analyzed with one-way ANOVA followed by the Tukey-Kramer post hoc test. Statistical differences are shown as *p< 0.05, **p<0.01, compared with non-treated control.

Transcription of *GPR43* was significantly increased in cells treated with 1.0 μM of GPR43 agonist, (S)-2-(4-chlorophenyl) -3, 3-dimethyl-N-(5-phenylthiazol-2-yl) butanamide [27], as well as acetic acid (Fig 4A). Transcriptions of the *mef2a*, *mb*, *cycs*, and *sdha* genes were also significantly increased by GPR43 agonist and acetic acid (Fig 4B). Furthermore, protein expressions of pAMPK, myoglobin, MEF2A, PGC-1α, NFATc1, and CaMKKβ were also up-regulated upon treatment with the GPR43 agonist and acetic acid (Fig 4C). Phosphorylated NFATc1 was decreased significantly with those treatments (Fig 4C).

### Effect of acetic acid on the induction of [Ca^2+^]_i_ influx in L6 cells upon *GPR43* silencing

**Fig 5:**
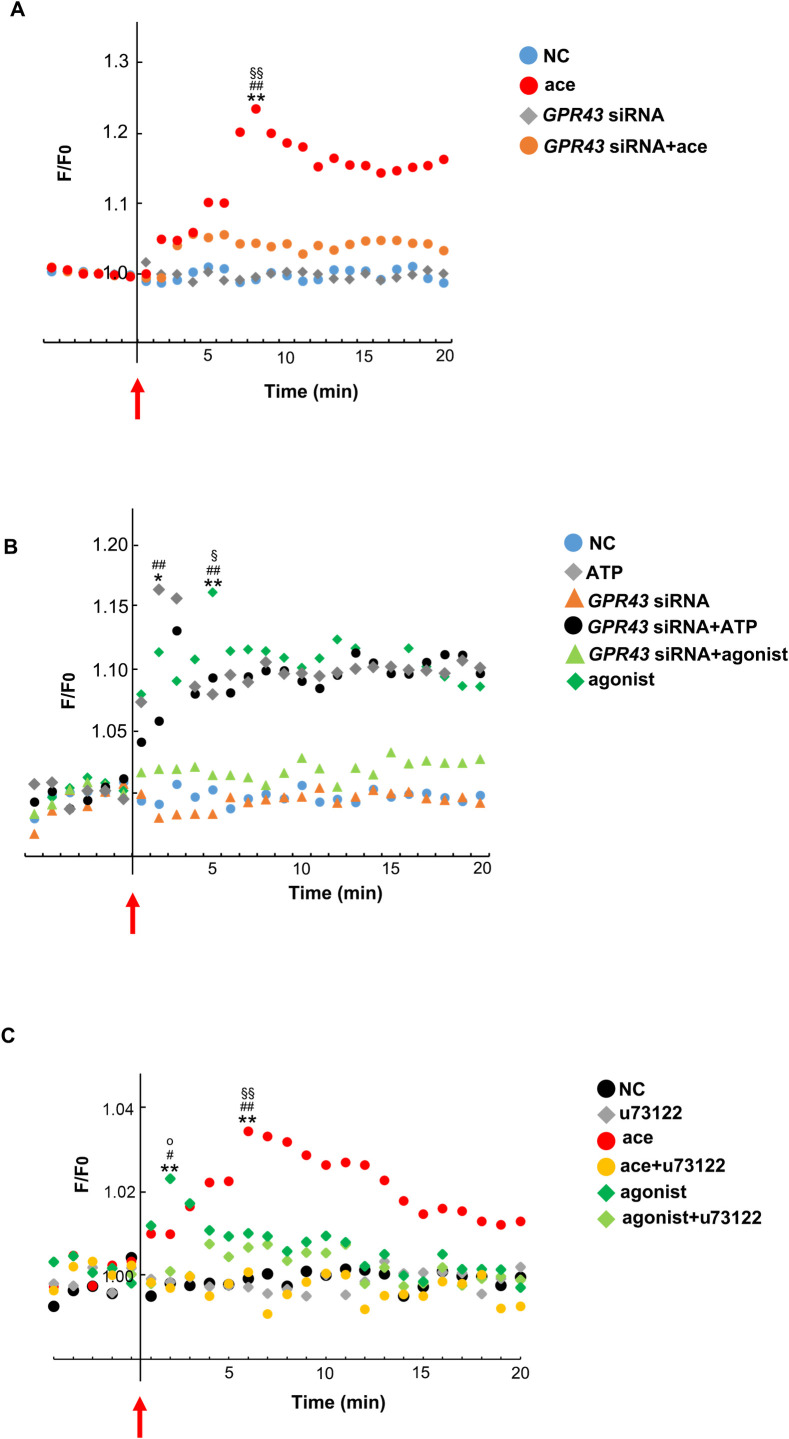
Effects of acetic acid, GPR43 agonist, *GPR43* silencing, and PLC inhibitor on the induction of [Ca^2+^]i influx in L6 cells. Changes in the [Ca^2+^]_i_ influx were measured in response to 0.5 mM acetic acid (A), 25 μM ATP, or 1.0 μM GPR43 agonist (B) in *GPR43*-siRNA transfected or non-transfected L6 cells as described in Materials and Methods. [Ca^2+^]_i_ changes were measured in response to acetic acid and agonist of GPR43 in L6 cells treated with or without PLC inhibitor, u73122, for 24 h (C). Arrow indicates addition of the stimulus. NC: Non-stimulated condition. Data shown are average values of 3 to 7 independent experiments. Peaks of average values for each treatment were analyzed with one-way ANOVA followed by Tukey-Kramer post hoc test. Statistical differences are shown as (A) **p< 0.01, compared with NC; ^##^p < .0.01, compared with *GPR43* siRNA; ^§§^p< 0.01, compared with *GPR43* siRNA + ace, (B) *p< 0.05, **p< 0.01, compared with NC; ^##^p < .0.01, compared with *GPR43* siRNA; ^§^p< 0.05, compared with *GPR43* siRNA + agonist, and (C) **p< 0.01, compared with NC; ^#^p< 0.05, ^##^p< 0.01, compared with u73122; ^§§^p< 0.01, compared with ace + u73122; ^o^p< 0.05, compared with agonist + u73122.

In order to examine the physiological functions of GPR43, induction of [Ca^2+^]_i_ influx was analyzed in the *GPR43*-silenced L6 cells upon the treatment with acetic acid and GPR43 agonist [24]. Intracellular [Ca^2+^]_i_ influx induced by ATP was not changed in cells transfected *GPR43*-siRNA. However, the [Ca^2+^]_i_ influx induced both by acetic acid and the GPR43 agonist was completely inhibited by siRNA mediated silencing of *GPR43* (Fig 5A and 5B).

Induction of [Ca^2+^]_i_ influx via GPR activation is caused by the stimulation of PLC activity and production of inositol-1,4,5-triphosphates (IP_3_) [28,29]. The PLC inhibitor, u73122, suppressed the intracellular [Ca^2+^]_i_ influx induced by acetic acid and GPR43 agonist (Fig 5C).

### Inhibition of PLC suppresses the phosphorylation of AMPK

**Fig 6:**
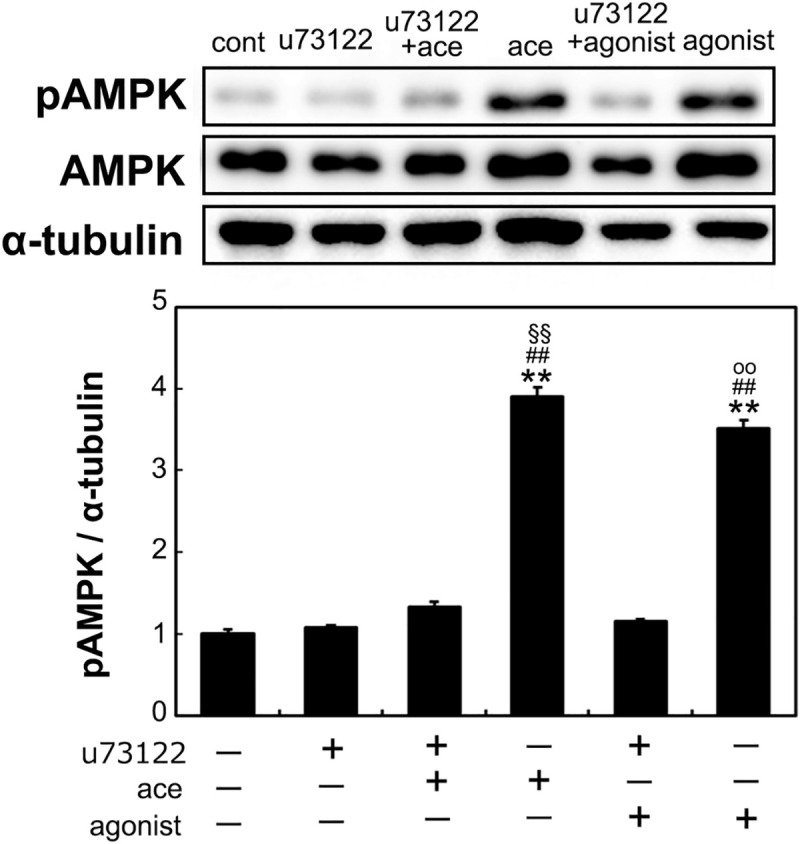
Effect of PLC inhibitor on the phosphorylation of AMPK in L6 myotube cells. Phosphorylated AMPK was analyzed in the L6 cells treated with 0.5 mM acetic acid or 1.0 μM GPR43 agonist for 30 min in the presence or absence of 1.0 μM PLC inhibitor, u73122, which was preincubated for 30 min, by western blotting as described in Materials and Methods. Multiple comparisons were analyzed with one-way ANOVA followed by the Tukey-Kramer post hoc test. Statistical differences are shown as **p< 0.01, compared with non-treated control; ^##^p< 0.01, compared with u73122; ^§§^p< 0.01, compared with u73122 + ace; ^oo^p< 0.01, compared with u73122 + agonist.

Acetic acid stimulates phosphorylation of AMPK. However, treatment with PLC inhibitor abolished the phosphorylation. Agonist of GPR43 also induced the phosphorylation of AMPK, which was diminished by the treatment of the PLC inhibitor (Fig 6).

### Association of calmodulin (CaM) and calcineurin with the increase of intracellular calcium levels upon acetic acid treatment

**Fig 7:**
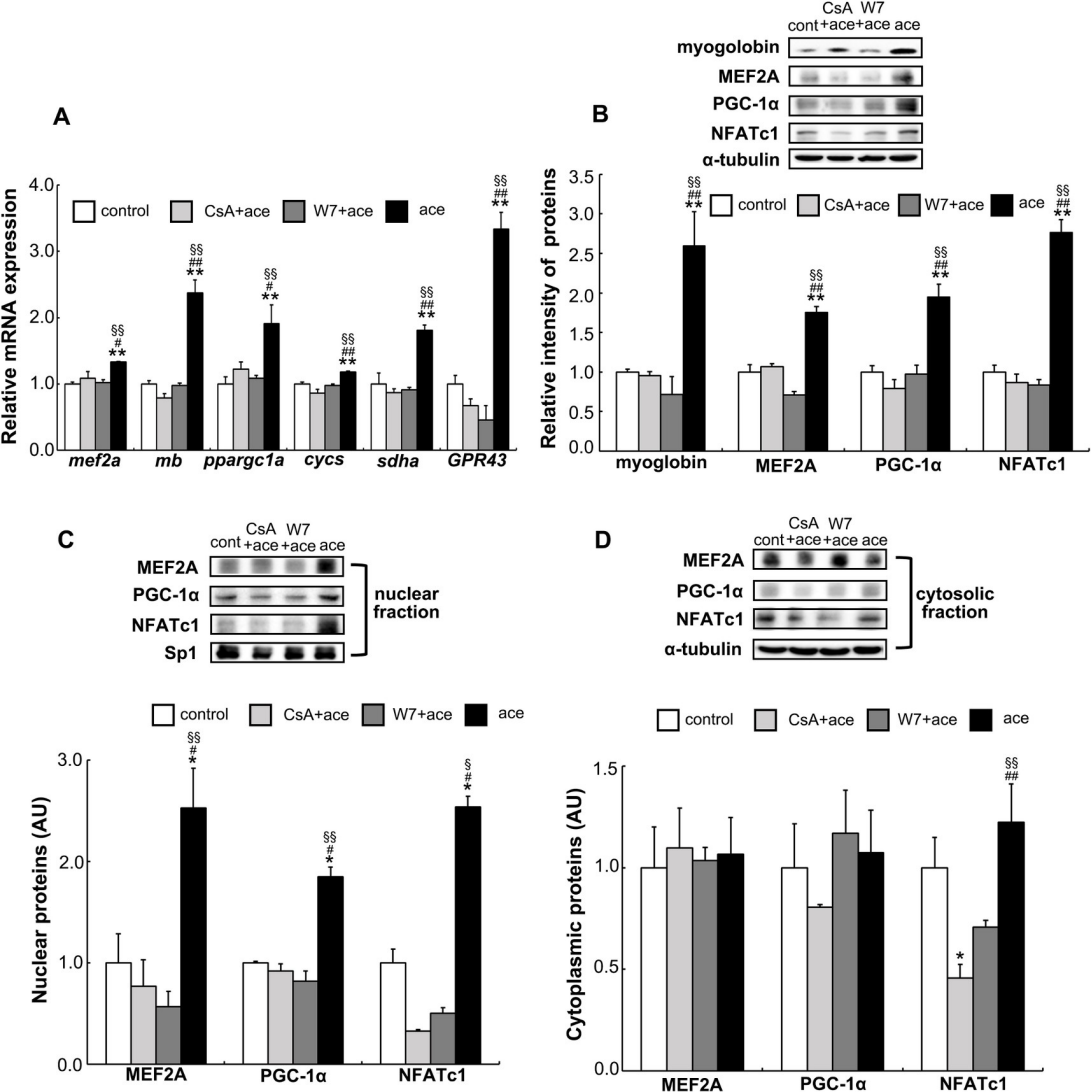
Effect of the calcineurin and CaM inhibitors on the expressions of genes and proteins by acetic acid treatment. Total RNA and proteins, along with the nuclear and cytoplasmic proteins, were extracted from L6 myotube cells that treated or untreated with 0.5 mM acetic acid for 30 min in the presence or absence of 10 μM CsA or 1.0 μM W7 preincubated for 1 h. (A) Expressions of *mef2a*, *mb*, *ppargc1a*, *cycs*, *sdha*, and *GPR43* genes were analyzed by real-time PCR as described in Materials and Methods. (B) Myoglobin, MEF2A, PGC-1α, and NFATc1 proteins were analyzed by western blotting as described in Materials and Methods. Nuclear (C) and cytosolic fractions (D) were separated and analyzed for the levels of MEF2A, PGC-1α, and NFATc1 proteins by western blotting. Each bar represents the mean ±SE (n = 3–6). Multiple comparisons were analyzed with one-way ANOVA followed by the Tukey-Kramer post hoc test. Statistical differences are shown as *p< 0.05, **p< 0.01, compared with control; ^#^p< 0.05, ^##^p< 0.01, compared with CsA + ace; ^§^p< 0.05, ^§§^p< 0.01, compared with W7 + ace.

Calcium/ CaM enhances the activation of calcineurin and stimulates de-phosphorylation of NFATc1, following an increase in the transactivation of target slow-twitch muscle fiber genes [26,30,31]. Calcineurin inhibitor, cyclosporine A (CsA), and CaM inhibitor, W7, completely suppressed the genes *mef2a*, *mb*, *ppargc1a*, *cycs*, *sdha*, and *GPR43*; and proteins such as myoglobin, MEF2A, PGC-1α, and NFATc1 that increased with the acetic acid treatment (Fig 7A and 7B). The expression levels of MEF2A, PGC-1α, and NFATc1 proteins in the nuclear fraction were also decreased because of the treatment with those inhibitors (Fig 7C). Contrastingly, MEF2A and PGC-1α proteins in the cytosolic fraction remained unaffected, though the NFATc1 expression was significantly decreased upon the treatment with CsA and W7 in cytosolic as well as nuclear fractions (Fig 7D).

### GPR43 is involved with mitochondrial proliferation

**Fig 8:**
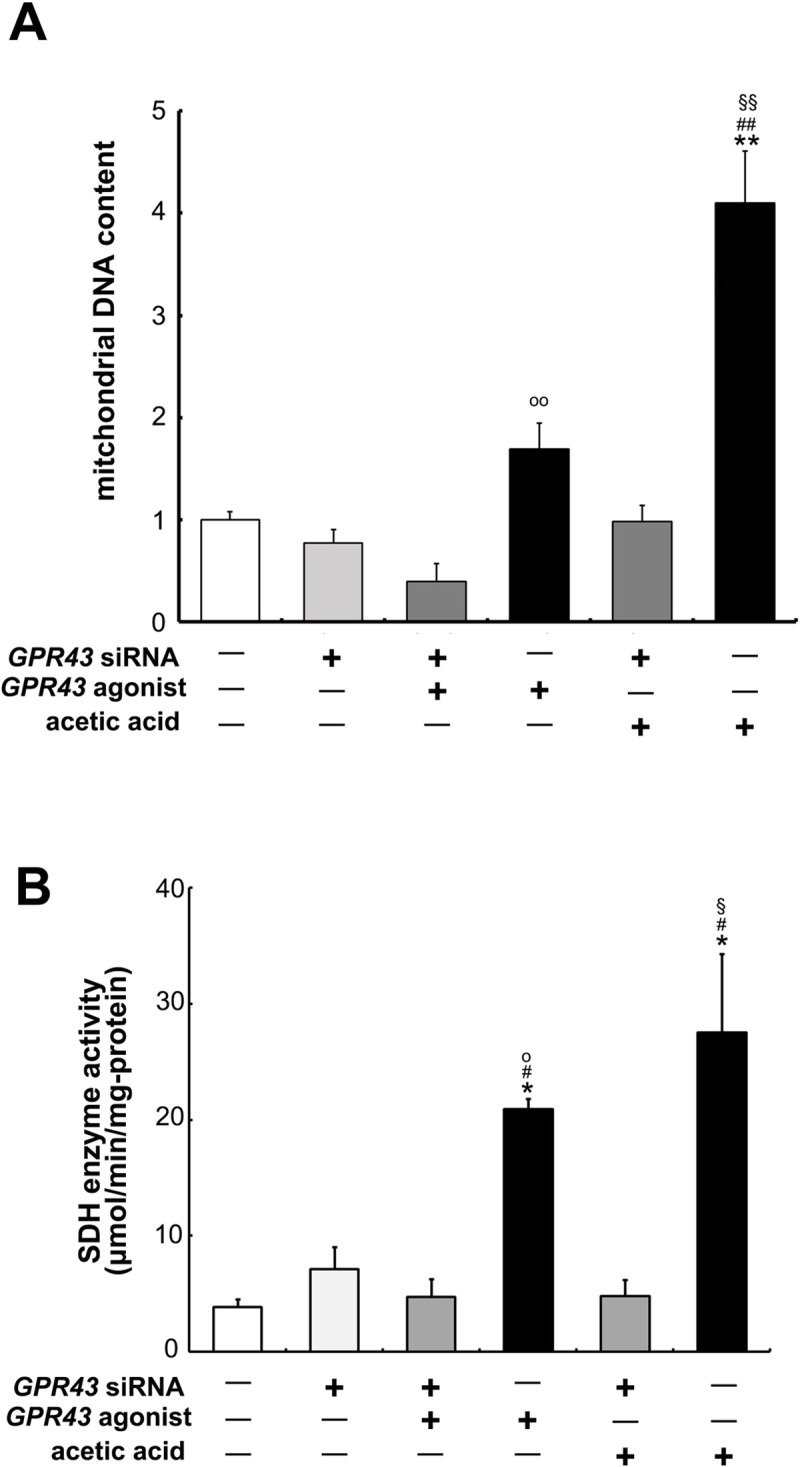
Proliferation of mitochondria by the treatment of acetic acid or GPR43 agonist through the activation of GPR43. (A) Genomic DNA was extracted from L6 cells that were transfected or non-transfected with *GPR43-* siRNA after the treatment or no treatment of 0.5 mM acetic acid or 1.0 μM GPR43 agonist for 30 min. Real-time PCR analysis was carried out for the determination of *mt-Nd1* level in L6 myotube cells. (B) SDH activity (nmol/min/mg of protein) in L6 cells of same condition with (A) was measured by consumption rate of DCPIP as described in Materials and Methods. Each bar represents the mean ± SE (n = 3–6). Multiple comparisons were analyzed with one-way ANOVA followed by the Tukey-Kramer post hoc test. Statistical differences are shown as *p< 0.05, **p< 0.01, compared with non-treated control; ^#^p< 0.05, ^##^p< 0.01, compared with *GPR43* siRNA; ^§^p< 0.05, ^§§^p< 0.01, compared with *GPR43* siRNA + ace; ^o^p< 0.05, ^oo^p< 0.01, compared with *GPR43* siRNA + agonist.

Relative quantity of mitochondrial DNA (mtDNA) and SDH activity, which is a mitochondrial respiratory enzyme, were significantly increased by the treatment of acetic acid and GPR43 agonist compared with the untreated control cells. However, transfection of *GPR43*-siRNA completely suppressed both the mtDNA levels and SDH activity in the cells (Fig 8A and 8B).

## Discussion

The GPR43 receptor is coupled to the Gq/_11_ protein that induces the calcium influx upon activation [12–14]. And it is activated by acetic acid and induces Ca^2+^ influx in transfected cells [12]. It has been reported that *GPR43* is expressed in the adipose tissue, intestinal, and immune cells, and activation of GPR43 leads to the suppression of fat accumulation, increase of GLP-1 secretion, and improves insulin signaling, however, the expression and the function of GPR43 in skeletal muscles have not been fully investigated [2,32,33]. SCFAs generated in the gut by microbes work as endogenous ligands for the GPR43 and GPR41 and act as signaling molecules in the tissues [12,13,34–36]. Acetic acid has much more affinity with GPR43 than that with GPR41 [13]. It was shown in our previous study that administered acetic acid functions as an activator of AMPK and accelerates mitochondrial lipid oxidation in skeletal muscles via AMP generation [6]. Dietary acetic acid may play a role as a GPR43 ligand. In this study, we showed that *GPR43* is expressed in L6 skeletal muscle cells and is activated by the treatment of acetic acid (Fig 1B).

GPR43 has been reported to be coupled to the Gq and Gi/o protein families; and through the intracellular signaling pathway, it elevates the generation of signaling molecules such as IP_3_ and intracellular Ca^2+^, and inhibits cAMP accumulation [37,38]. In skeletal muscles, a rise in the intracellular calcium influx resulting from motor activation plays key role in contractile activity-dependent expression as well as fiber type-specific gene expression [30]. A number of Ca^2+^ sensitive target genes have been identified in the skeletal muscles [30,39,40]. The key signaling pathway downstream to the elevation in intracellular calcium that translates this signal into a transcriptional response includes the Ca^2+^/ CaM-dependent phosphatase calcineurin pathway [26,30]. Calcineurin is a serine/threonine phosphatase and is activated by the binding of Ca^2+^/CaM. The best-known transcriptional targets for calcineurin are members of the NFAT family. The NFAT transcription factor translocates to the nucleus upon de-phosphorylation by calcineurin [24,26], promotes binding to the nucleotide recognition sequence, and enhances the transcription of the target genes [41]. In the nucleus, NFAT protein interacts with target genes in conjunction with other transcriptional regulator, MEF2, binding sites for which are clustered in the promoter/enhancer regions controlling the transcription of genes encoding proteins of the slow-fiber program [42,43]. Upstream of *GPR43* gene contains NFAT recognition site [44]. In this study, it indicates that NFAT controls gene expression of *GPR43* as well as other muscle oxidative fiber genes.

MEF2 proteins are the transcription factors involved in skeletal muscle differentiation [26]. The Ca^2+^/CaM -dependent protein kinase (CaMK) is a potent activator of MEF2A activity [26,45,46]. Studies report that NFAT associates with MEF2 and recruits the p300 co-activator to the MEF2 target genes and serves to stabilize the NFAT-MEF2 complex. It was shown that the Ca^2+^/CaM-mediated activation of CaMK phosphorylates histone deacetylase 5 (HDAC5), which binds directly to the MEF2 proteins and represses the transcriptional activity, and the direct interaction of the Ca^2+^/CaM with the repressor core of HDAC5 releases HDAC5 from MEF2 proteins [26,47,48]. After this, the phosphorylated HDAC5 masks the nuclear localization sequence, exposes the nuclear export signal by binding of the 14-3-3 chaperone protein, and is subsequently sequestered in the cytosol [47,49]. Subsequently, the transcriptional adapter factor p300 can bind to MEF2 and acetylate the histone protein tails. It has been reported that PGC-1α gene expression is also regulated by calcium-signaling components in the skeletal muscle cells [50]. PGC-1α plays a key role in the regulation of mitochondrial biogenesis and oxidative metabolism. Promoter sequence of *PGC-1α* contains a MEF2 binding site and those expressions are regulated by mutual interactions [51,52].

Furthermore, the activities of PGC-1α and MEF2A are regulated by AMPK [53]. AMPK is known as the master regulator of metabolic homeostasis. Activation of AMPK also leads to an increased mitochondrial biogenesis in the skeletal muscles [25,52]. AMPK is involved in the long-term metabolic changes involving mitochondrial biogenesis and promotes an oxidative muscle phenotype [54]. AMPK is also activated by CaMKK that is activated by Ca^2+^/CaM [25,55,56]. In this study, it appears that acetic acid functions to activate AMPK through stimulation of calcium release via activation of GPR43. Our results show that MEF2A and PGC-1α genes’ and proteins’ expression levels are suppressed by the treatment of CsA and W7 (Fig 7). Acetic acid generated by microbial fermentation of the dietary fiber in gut could be an activator of GPR43 in skeletal muscles. The ingested acetic acid would also have the effect on the activation of GPR43 in skeletal muscles and may improve the muscle functioning of slow-twitch fiber [6].

In the previous study, it was presented that chronic intake of acetic acid has effects on lower lipid accumulation in adipose tissue, on suppression of lipogenesis in liver and of plasma triglyceride, and on improvement of glucose tolerance in type 2 diabetic rats [5,6]. Lu *et al*. showed that dietary supplementation of acetic acid protects high-fat diet induced obesity and increased expressions of *GPR43* and *41* in the adipose tissue [57]. Kimura *et al*. found that SCFAs, including acetic acid, activate GPR43 that expressed in the adipose tissue of mice and contribute to the regulation of energy expenditure and the suppression of fat accumulation [2]. Treatment of acetic acid stimulates fatty acid metabolism and glucose incorporation in L6 skeletal muscle cells through the mechanism of AMPK activation [9]. In this study, it appears that acetic acid activates GPR43 and its activation leads to the stimulation of AMPK and the increase of genes expression relating to slow-twitch fiber in L6 skeletal muscle cells. These effects of acetic acid may lead to postprandial glucose uptake and lipid-lowering effect by vinegar ingestion. Actually in human study, it was shown that daily intake of vinegar, which contains 750-1500mg acetic acid, has effects on suppressions of body weight, BMI, visceral fat area, waist circumference, and serum triglyceride [8]. Furthermore, acute ingestion of vinegar, which is containing 6% acetic acid, has effects on postprandial glucose uptake in forearm muscle and on lipid-lowering effect, suggesting improvement of insulin sensitivity [58]. In conclusion, our data indicate that acetic acid activates GPR43 in L6 skeletal muscles and stimulates gene expressions associated with slow-twitch fiber by regulating the functions of NFATc1, MEF2A, PGC-1α, and CaMKK through calcium-signaling. Daily intake of acetic acid may prevent obesity and improve skeletal muscle functioning during ageing process via the function of GPR43 and AMPK. It would be needed further investigation in human skeletal muscle cells.

## Supporting information

S1 Fig Effect of PLC inhibitor, YM-254890, on the phosphorylation of AMPK in L6 myotube cells. Phosphorylated AMPK was analyzed in the L6 cells treated with 0.5 mM acetic acid or 1.0 μM GPR43 agonist for 30 min in the presence or absence of 1.0 μM PLC inhibitor, YM-254890, which was preincubated for 5 min, by western blotting as described in Materials and Methods. Multiple comparisons were analyzed with one-way ANOVA followed by the Tukey-Kramer post hoc test. Statistical differences are shown as *p< 0.05, **p< 0.01, compared with non-treated control; ^##^p< 0.01, compared with YM-254890; ^§§^p< 0.01, compared with YM-254890 + ace; ^oo^p< 0.01, compared with YM-254890 + agonist.
(TIF)

S1 Raw Image Supplementary file of Fig 1. (TIF)

S2 Raw Image Supplementary file of Fig 3C. (TIF)

S3 Raw Image Supplementary file of Fig 3D. (TIF)

S4 Raw Image Supplementary file of Fig 4C. (TIF)

S5 Raw Image Supplementary file of Fig 6. (TIF)

S6 Raw Image Supplementary file of Fig 7B. (TIF)

S7 Raw Image Supplementary file of Fig 7C and 7D. (TIF)

S8 Raw Image Supplementary file of S1 Fig. (TIF)
