# Supplementary figures and images for: Acetic acid stimulates G-protein-coupled receptor GPR43 and induces intracellular calcium influx in L6 myotube cells
Source: PLoS One. 2020 Sep 30;15(9):e0239428. doi: 10.1371/journal.pone.0239428 (PMC7526932; doi:10.1371/journal.pone.0239428)

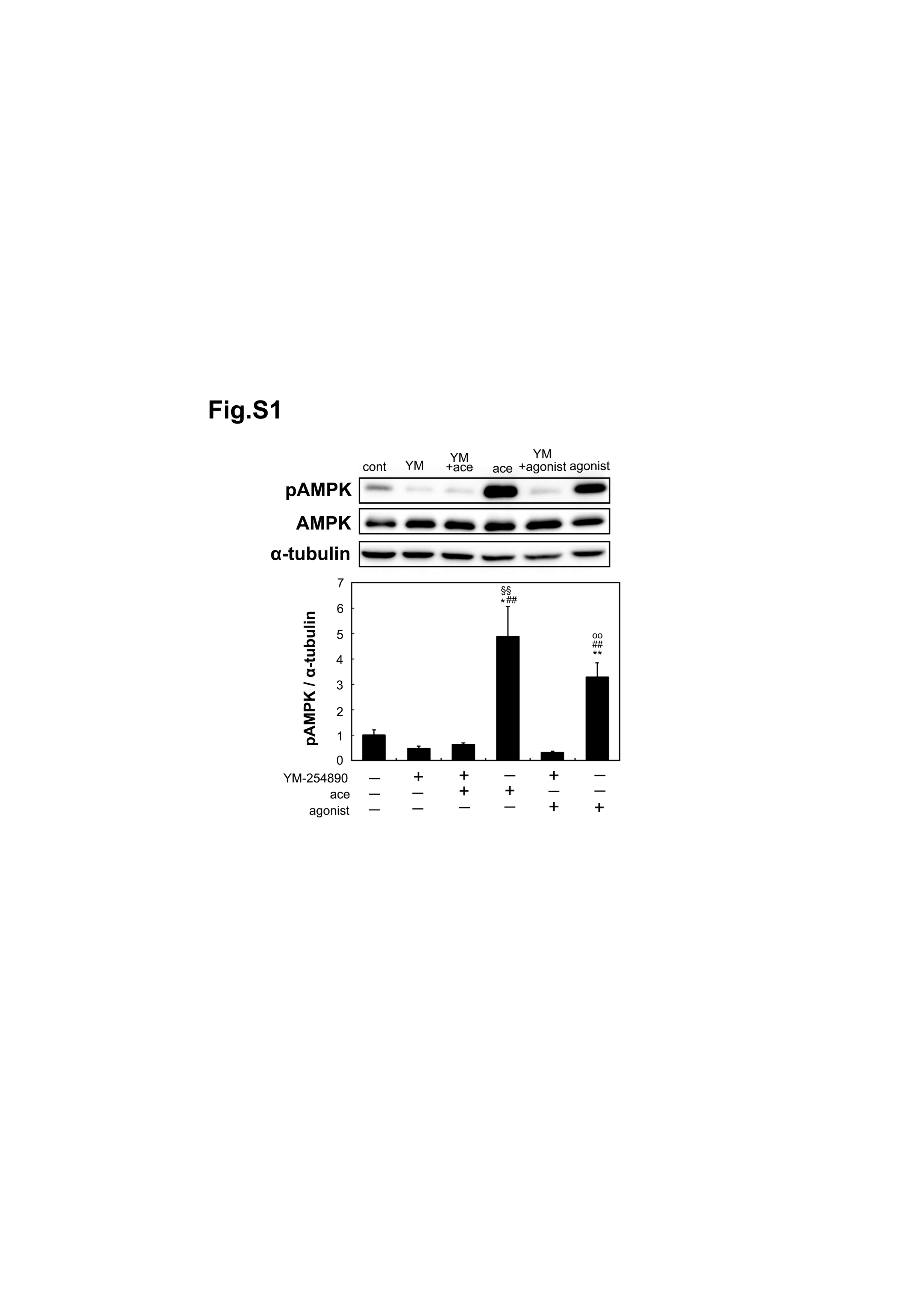

Supplement: S1 Fig — Phosphorylated AMPK was analyzed in the L6 cells treated with 0.5 mM acetic acid or 1.0 μM GPR43 agonist for 30 min in the presence or absence of 1.0 μM PLC inhibitor, YM-254890, which was preincubated for 5 min, by western blotting as described in Materials and Methods. Multiple comparisons were analyzed with one-way ANOVA followed by the Tukey-Kramer post hoc test. Statistical differences are shown as *p< 0.05, **p< 0.01, compared with non-treated control; ##p< 0.01, compared with YM-254890; §§p< 0.01, compared with YM-254890 + ace; oop< 0.01, compared with YM-254890 + agonist. (TIF) [file pone.0239428.s001.tif]

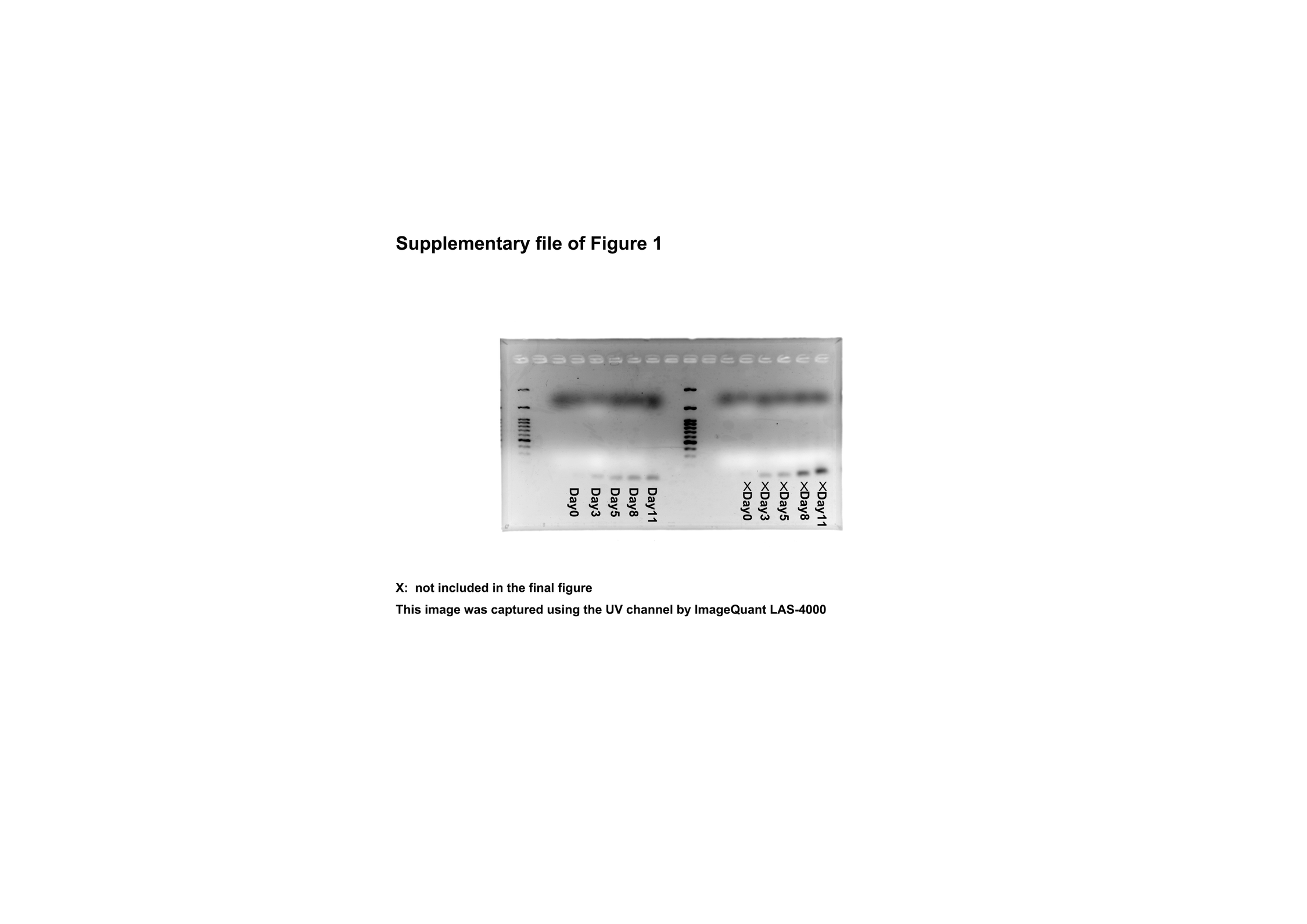

Supplement: S1 Raw Image — (TIF) [file pone.0239428.s002.tif]

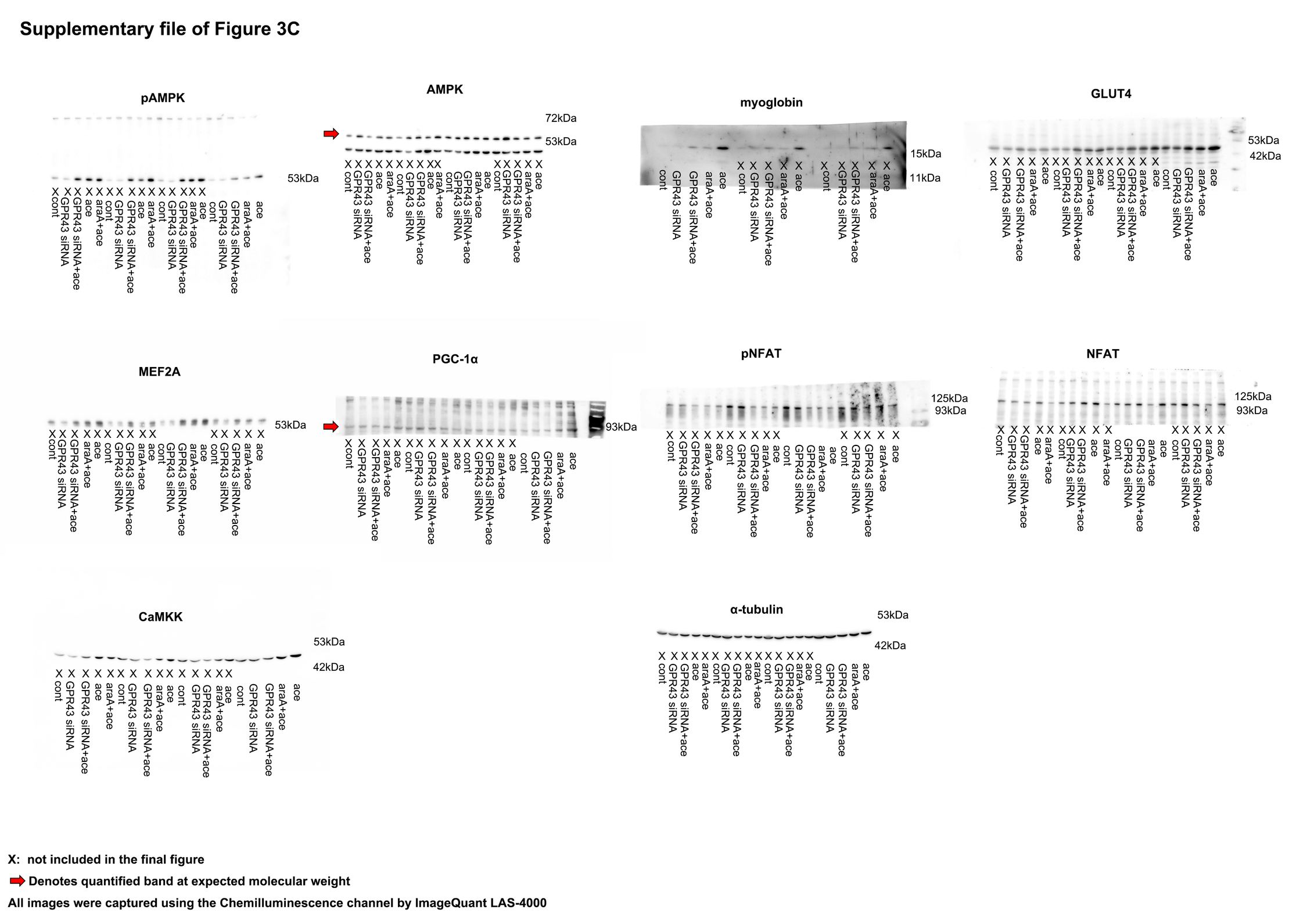

Supplement: S2 Raw Image — (TIF) [file pone.0239428.s003.tif]

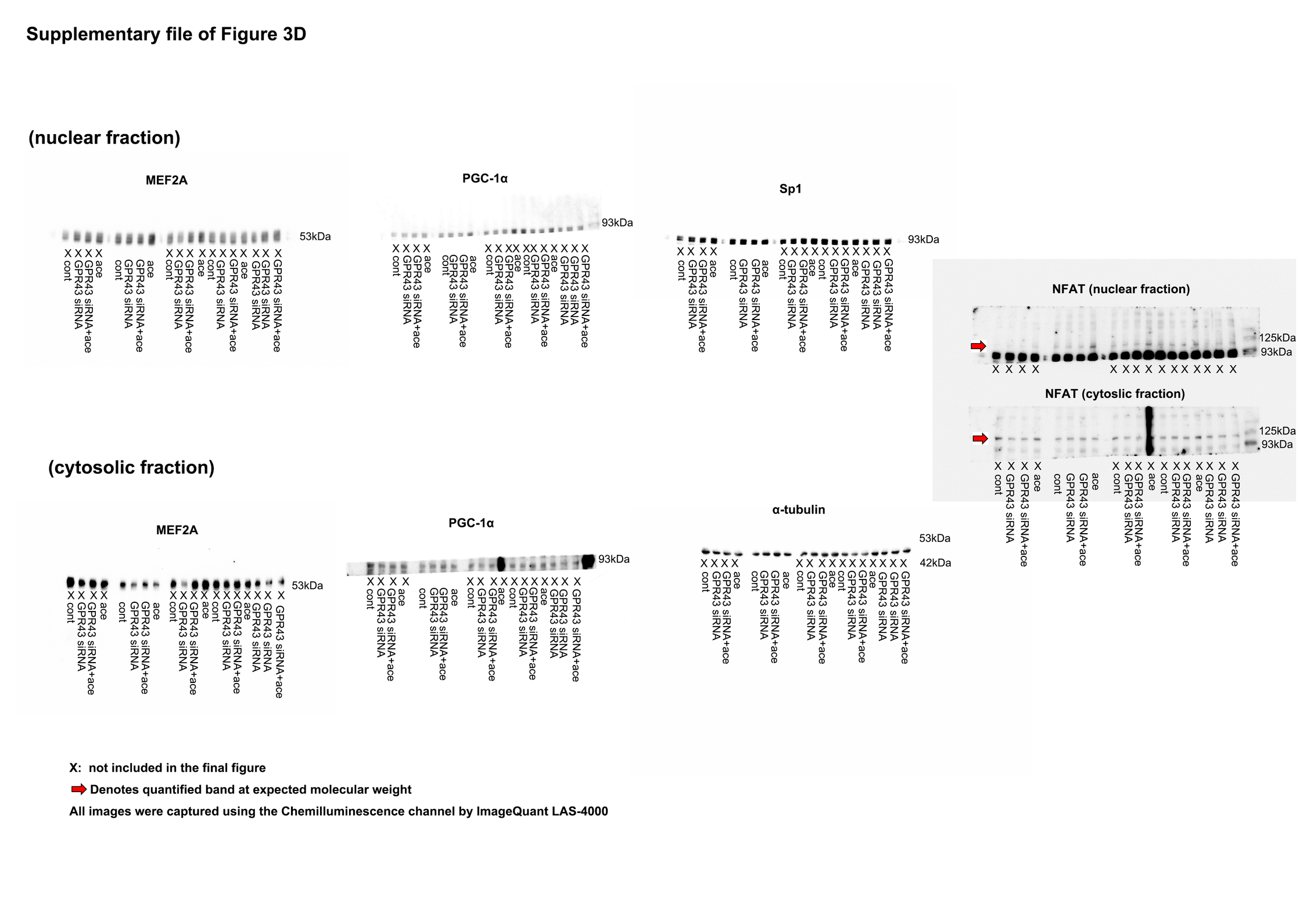

Supplement: S3 Raw Image — (TIF) [file pone.0239428.s004.tif]

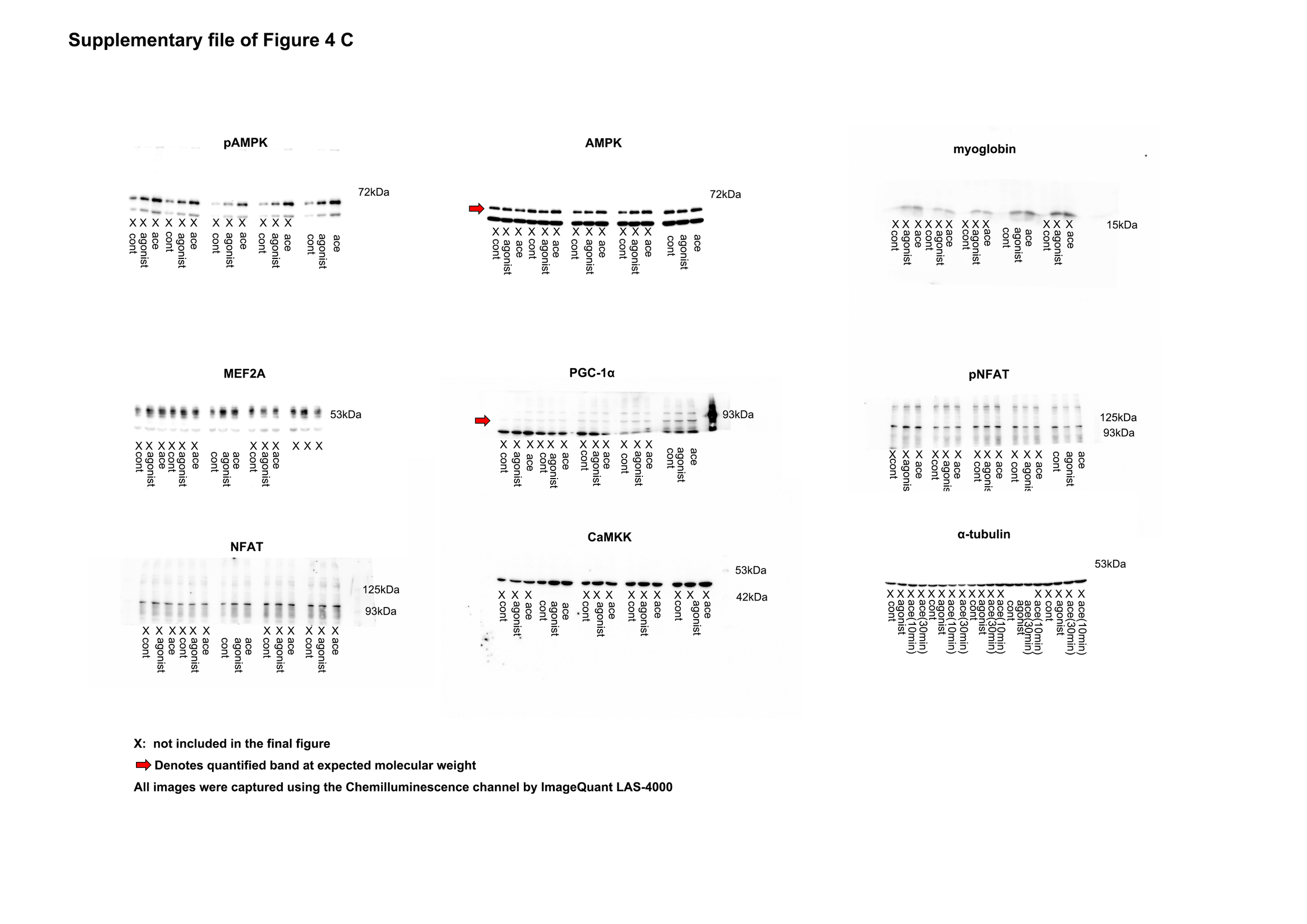

Supplement: S4 Raw Image — (TIF) [file pone.0239428.s005.tif]

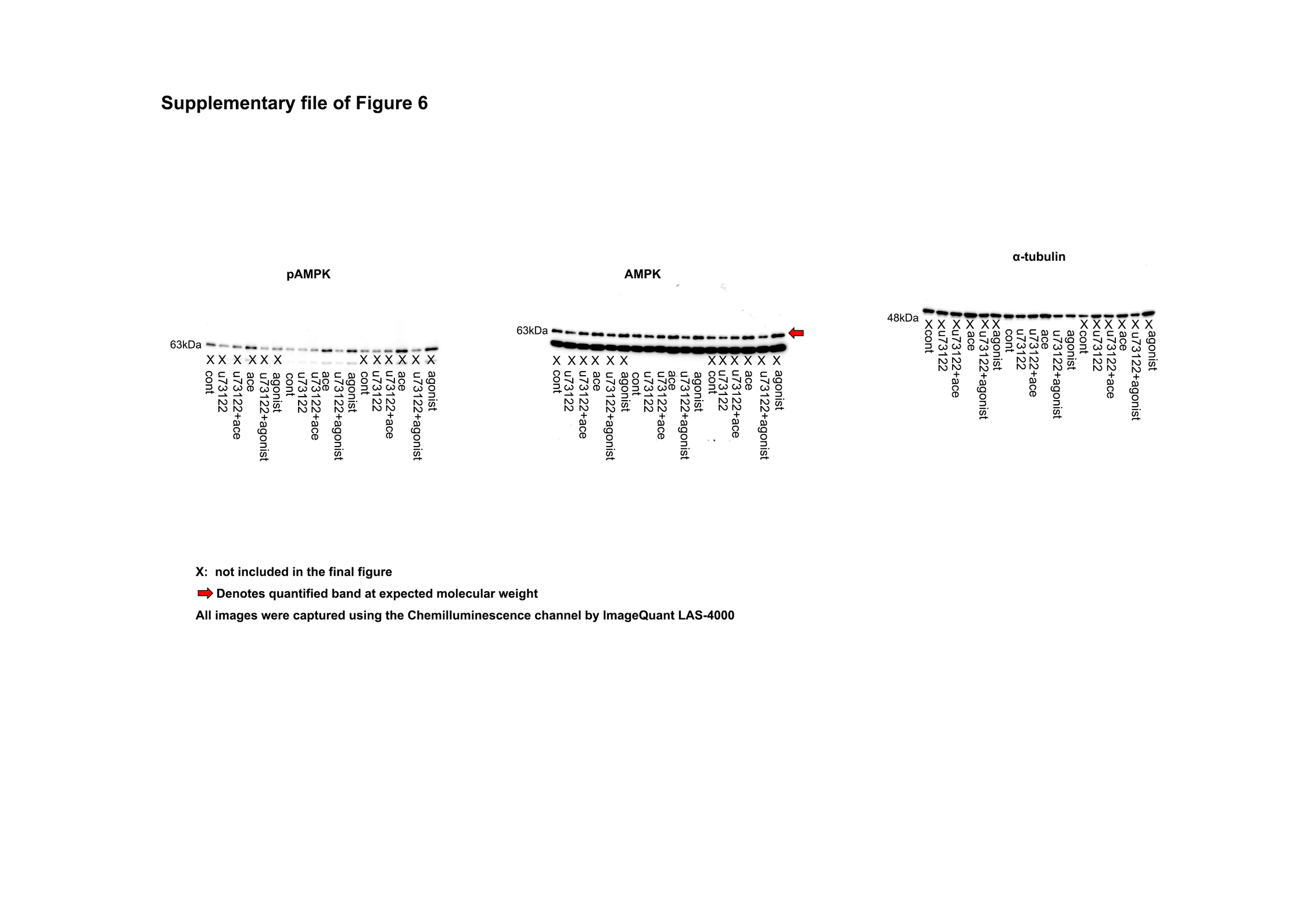

Supplement: S5 Raw Image — (TIF) [file pone.0239428.s006.tif]

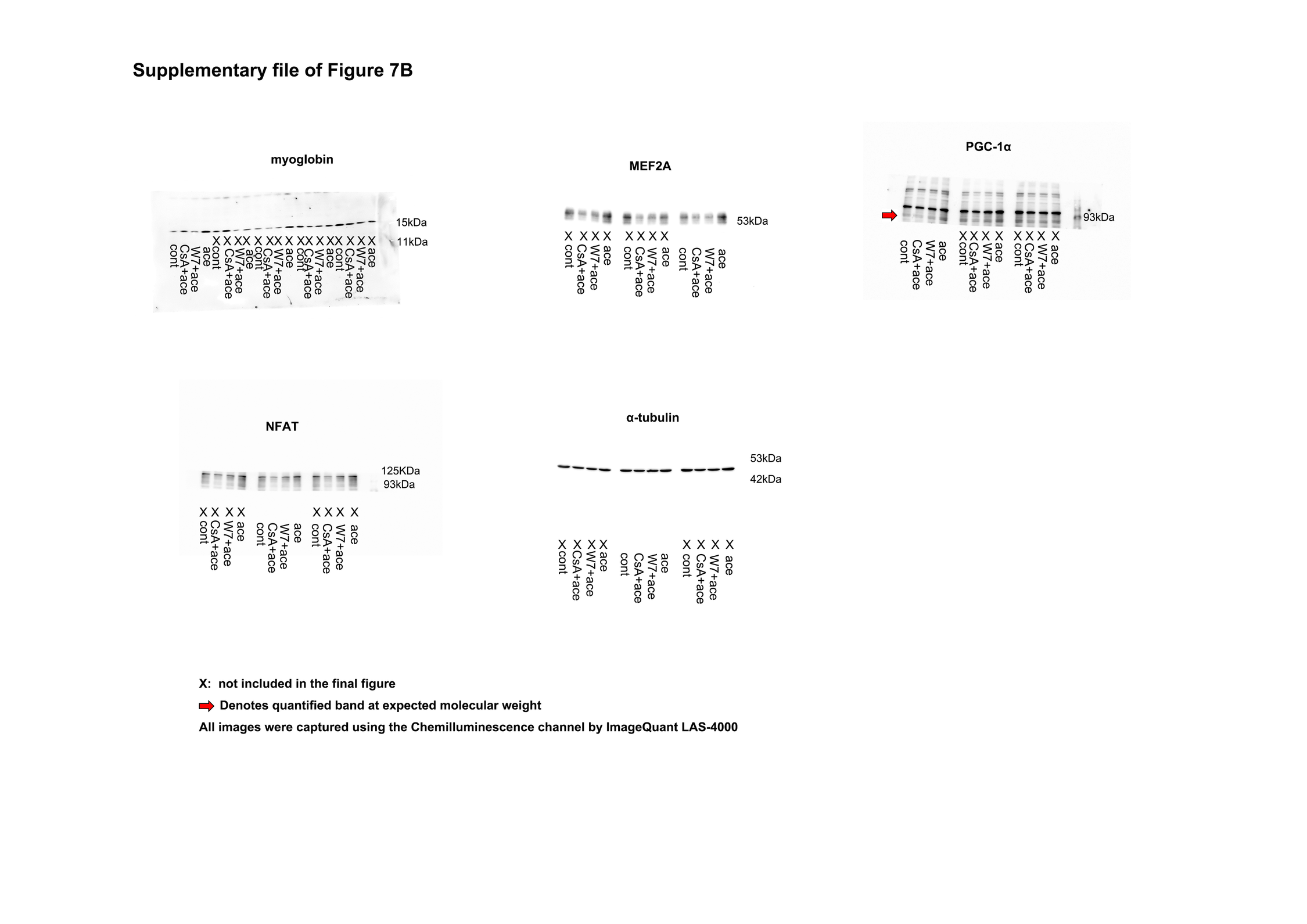

Supplement: S6 Raw Image — (TIF) [file pone.0239428.s007.tif]

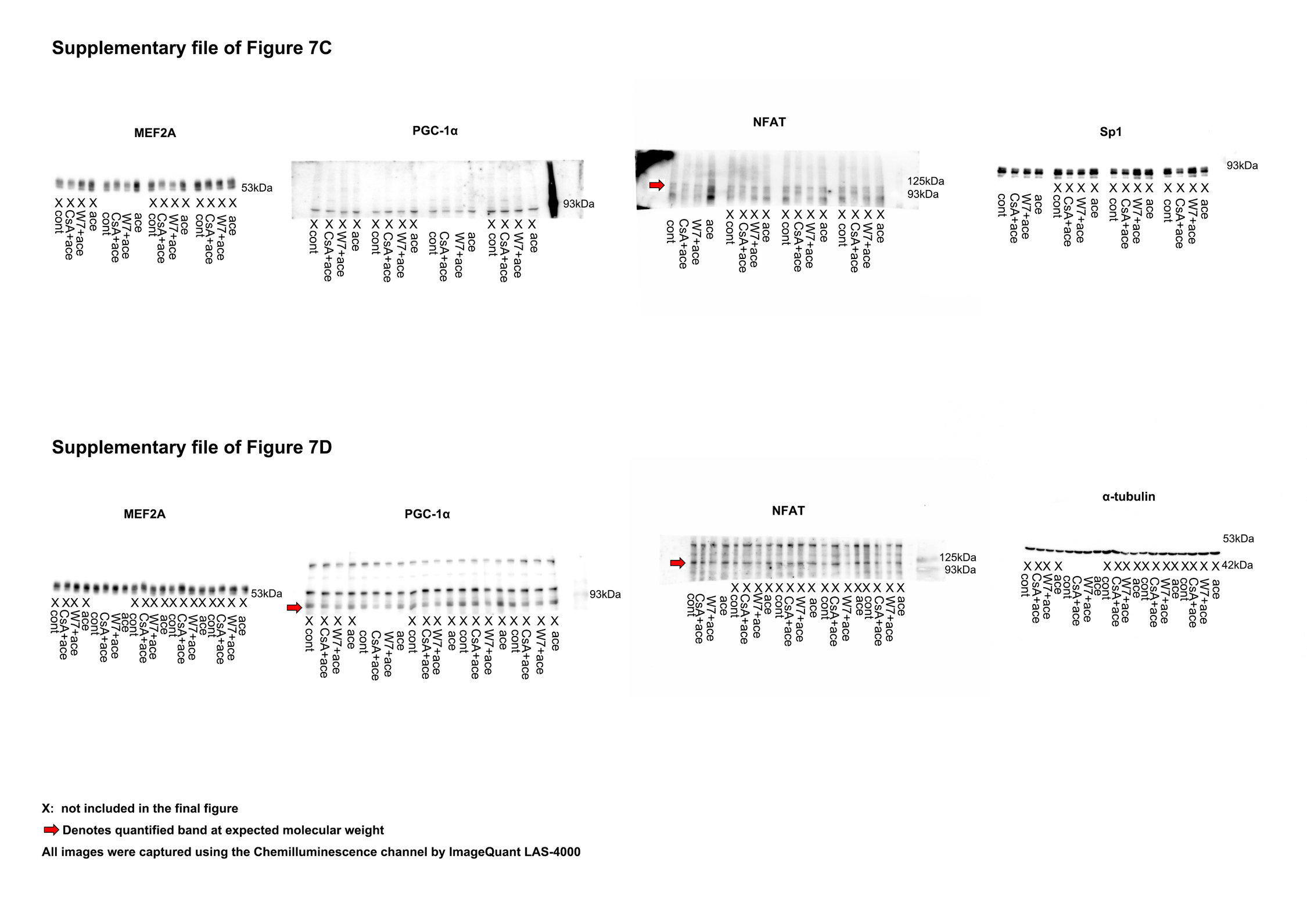

Supplement: S7 Raw Image — (TIF) [file pone.0239428.s008.tif]

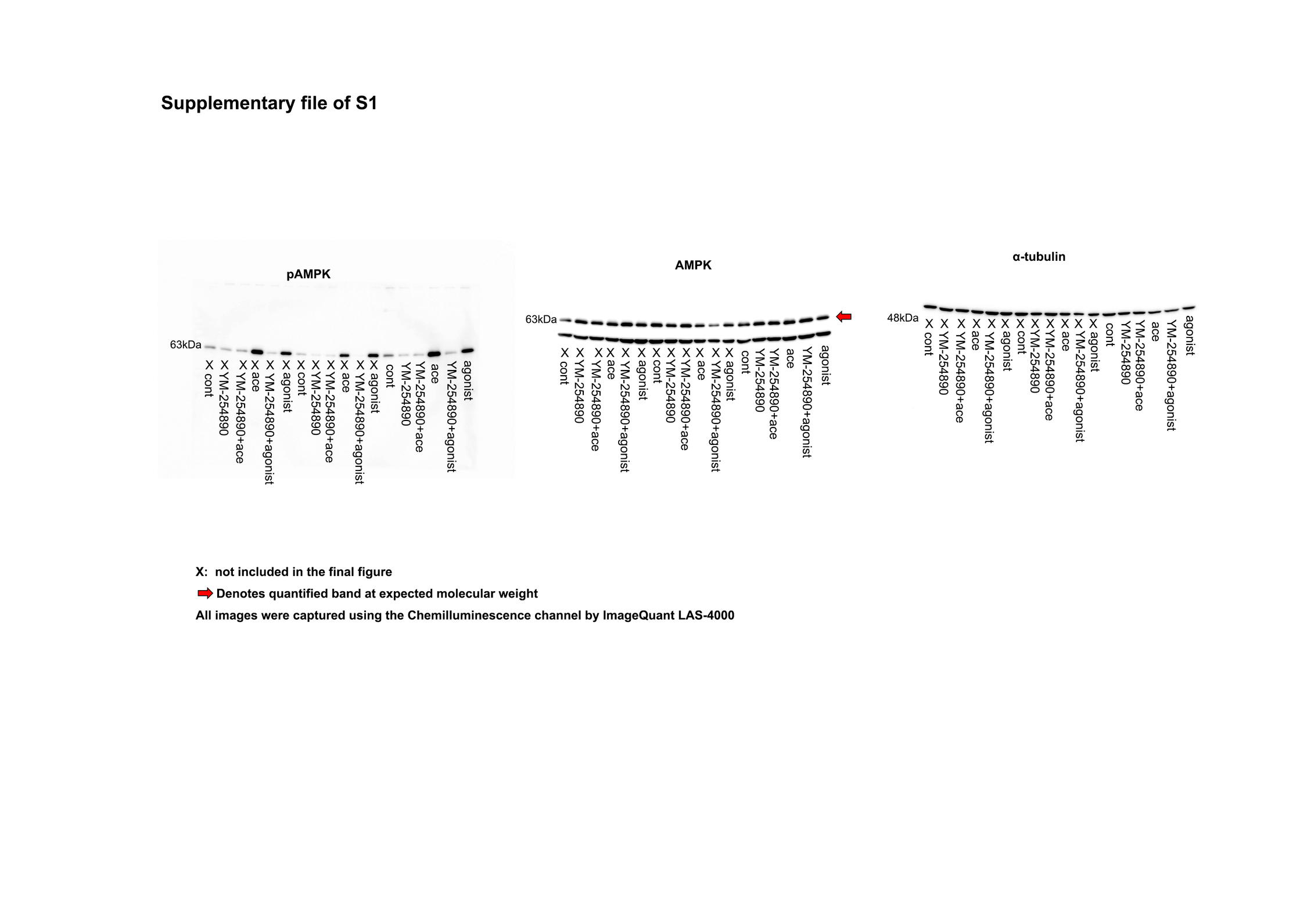

Supplement: S8 Raw Image — (TIF) [file pone.0239428.s009.tif]
